# What a Volcano Can Do

**Published:** 1884-07

**Authors:** 


### WHAT A VOLCANO CAN DO.

COTAPAXI, in 1738, threw its fiery rockets 3,000 feet above its crater, while in 1754 the blazing mass, struggling for an outlet, roared so that its awful voice was heard at a distance of more than 600 miles. In 1767 the crater of Tunguragua, one of the great peaks of the Andes, flung out torrents of mud, which dammed up the rivers, opened new lakes, and, in valleys 1,000 feet wide, made deposits 600 feet deep. The stream from Vesuvius, which, in 1837, passed through Torre del Greco, contained 33,000,000 cubic feet of solid matter, and in 1793, when Torre del Greco was destroyed a second time, the mass of lava amounted to 45,000,000 cubic feet. In 1760 Ætna poured forth a flood which covered 84 square miles of surface, and measured nearly 1,000,000,000 cubic feet. On this occasion the sand and scoria formed the Monte Rosini, near Nicholosa, a cone of two miles in circumference, and 4,000 feet high. The stream thrown out by Ætna in 1810 was in motion at the rate of a yard a day for nine months after the eruption; and it is on record that the lava of the same mountain, after a terrible eruption, was not thoroughly cool and consolidated for 10 years after the event. In the eruption of Vesuvius, A. D. 79, the scoria and ashes vomited forth far exceeded the entire bulk of the mountain; while in 1660 Ætna disgorged more than twenty times its own mass. Vesuvius has sent its ashes as far as Constantinople, Syria and Egypt; it hurled stones eight pounds in weight to Pompeii, a distance of six miles where similar masses were tossed up 2,000 feet above the summit. Cotapaxi has projected a block of 100 cubic yards in volume a distance of nine miles; and Sumbawa, in 1815, during the most terrible eruption on record, sent its ashes as far as Java, a distance of 300 miles of surface, and, out of a population of 12,000, only 20 escaped.

